# Fetomaternal outcomes in pregnant women with hepatitis E infection; still an important fetomaternal killer with an unresolved mystery of increased virulence in pregnancy

**DOI:** 10.4274/tjod.15045

**Published:** 2017-06-15

**Authors:** Namrata Kumar, Vinita Das, Anjoo Agarwal, Amita Pandey, Smriti Agrawal

**Affiliations:** 1 King George’s Medical University, Department of Obstetrics and Gynecology, Lucknow, India

**Keywords:** Encephalopathy, hepatitis B, hepatitis E, jaundice, Pregnancy

## Abstract

**Objective:**

Hepatitis is a prevalent infection in developing countries. While hepatitis B and C are deepening their roots in the developed world, hepatitis A and E are common in the developing world. The uniqueness of hepatitis is in its transformation from a relatively self-limiting disease in the non-pregnant state, to a highly virulent disease during pregnancy.

**Materials and Methods:**

This retrospective observational study was conducted in the Department of Obstetrics and Gynecology, King George’s Medical University, Lucknow, for a period of six months from June 2016 to November 2016 [probably during an endemic peak of hepatitis E virus (HEV)] to observe the clinical outcomes in HEV-infected pregnant women.

**Results:**

A total of 32 anti-HEV immunoglobulin M-positive pregnant women were included, and fetomaternal outcomes were analyzed. Hepatitis E positivity was significantly associated with maternal mortality, intrauterine demise with prematurity, and premature rupture of membranes was the most common fetal complication noted.

**Conclusion:**

The difference in extent of virulence of infection and variations in maternal morbidity, mortality, and rates of intrauterine demise, signify the presence of some factors that play a role and need to be further studied and evaluated.

## PRECIS:

Hepatitis E in pregnancy is a virulent disease with high fetal and maternal morbidity and mortality. The burden of the disease can be reduced by providing better sanitary services and clean drinking water to pregnant women.

## INTRODUCTION

Jaundice in pregnancy is a known high-risk factor that increases fetomaternal morbidity and mortality. While hepatitis B and C are deepening their roots in the developed world, hepatitis A and E are still more common in the developing world. The uniqueness of hepatitis E lies in its transformation from a relatively self-limiting disease in the non-pregnant state to a highly virulent disease during pregnancy. Hepatitis E virus (HEV) belongs to genus Hepevirus and family Hepeviridae. The RNA genome remains enclosed within a capsid composed of one or possibly two proteins, but many questions remain regarding its antigenicity.

Endemics usually occur during the rainy season. Once ingested, the virus first infects the liver followed by viremia and shedding in stool. Liver injury coincides with an elevation of transaminases and the appearance of anti-HEV immunoglobulin (Ig) M. The mechanisms behind its aggressive course during pregnancy are still not clearly understood. Clinical presentation varies from asymptomatic infection to anicteric, icteric, and fulminant hepatitis. Common presenting symptoms include yellowing of the eye and urine, fever, chills, anorexia, nausea, and abdominal pain. Aminotransferases are markedly elevated and may precede the onset of symptoms. Unlike hepatitis B virus (HBV) and hepatitis C virus (HCV), HEV infections are not known to cause cirrhosis or hepatocellular carcinoma.

Pregnant women are more susceptible to infection by HEV and progression to fulminant hepatic failure with high mortality rates and preterm deliveries. The disease is amenable to being prevented by a better sanitation check because the virus has a feco-oral route of transmission.

The present study was planned to evaluate the extent of maternal and fetal morbidity and mortality encountered due to hepatitis E in a tertiary care centre in North India.

## MATERIALS AND METHODS

This retrospective observational study was conducted in the Department of Obstetrics and Gynecology, King George’s Medical University, Lucknow, India, over a period of six months from June 2016 to November 2016, the endemic season for hepatitis E. All women with jaundice or history of jaundice in the present pregnancy who delivered during that period were recruited in the study. Jaundice was diagnosed by physical examination but a confirmation was made using liver function tests. The prevalence of jaundice in pregnancy was calculated along with percentages of hepatitis A, hepatitis B, hepatitis C positivity and hepatic encephalopathy. Hepatitis B and C positivity was diagnosed using and enzyme-linked immunosorbent assay and hepatitis A and E were confirmed through IgM positivity. Maternal and fetal outcomes were also noted from the labor registers and delivery records. The study was reviewed and given clearance by the institutional ethics committee.

## RESULTS

Total number of women who delivered during this six-month period was 3692, 177 (4.7%) of whom had jaundice at the time of delivery or during the antenatal period. The women with jaundice were studied in detail and data were classified patients who were HBV positive, hepatitis A virus (HAV) positive, HCV positive, HEV positive, and those who did not have positive viral markers but had jaundice due to other causes such as severe preeclampsia, sepsis, typhoid, dengue or no definite cause. The women who delivered were further classified by their age, antenatal care and registration, period of gestation at delivery, mode of delivery, antepartum or intrapartum fetal demise, preterm premature rupture of membranes, hepatic encephalopathy, and derangement in liver function test, platelets, and coagulation profile. The mean period of gestation at the time of onset of jaundice was 31.7±7.3 weeks.

The age of the patients ranged between 20-38 years with a mean age of 25 years. Table 1 shows the different causes of jaundice encountered in our study. The mode of delivery and fetomaternal outcomes were recorded as shown in Tables 2 and 3. A detailed description of the hepatitis E-positive pregnant women is presented in Table 4. About 90% of preterm deliveries were spontaneous, and 10% were medically induced due to comorbid indications.

## DISCUSSION

Viral hepatitis is one of the most common causes of jaundice encountered during pregnancy. Amongst all types of viral hepatitis, hepatitis E causes the most damage and is most prevalent in Asia and Africa^(1)^. The prevalence of the disease in the developed world is less and the difference is remarkable. Lachish et al.^(2)^ in a 10-year retrospective analysis found only fifteen pregnant women infected with HEV in Israel. Five (33%) patients in their series resulted in fulminant hepatitis, and two patients underwent urgent liver transplantation. They had no mortality among the mothers and fetuses, even in cases that resulted in fulminant liver failure. Other industrialized countries also rarely encounter autochthonous cases of hepatitis E in pregnancy^(3)^. We should realize that the scenario in developing countries like India, Pakistan^(4)^ and Bangladesh^(5)^ needs to be identified where both prevalence, morbidity, and mortality is high, and advanced modalities of treatment such as liver transplantation are not freely available. The high prevalence is also a constant threat for pregnant women travelling from industrialized countries^(6)^.

Disease outbreaks have shown to suddenly increase the number of maternal deaths as reported by Gurley et al.^(7)^ who reported that a sudden increase in jaundice deaths due to fulminant liver failure in pregnancy was retrospectively investigated and anti-HEV IgM positivity was seen in the neighbours of those who died. An accidental sewage contamination of municipal water led to maternal deaths, increased miscarriages, and perinatal mortality.

Contrary to its relatively benign course in the non-pregnant state, the infection takes a significantly virulent turn during pregnancy. Systematic reviews and meta-analyses have shown that patients in later gestations, particularly in the third trimester of pregnancy with HEV positivity or with fulminant hepatic failure, were significantly associated (p<0.05) with maternal mortality and intrauterine fetal death (27.0%)^(8)^.

HEV is chiefly an enterically transmitted virus, but other modes of transmission have also been proposed including person-to-person transmission, blood, and transplacental transmission^(9)^. Transplacental transmission is probably the cause behind the high incidence of abortion, preterm labor, and intrauterine demise associated with the disease. Interestingly, HEV replication has also been reported to occur in extra-hepatic sites such as the placenta^(10)^.

Various Indian studies have confirmed the deadly nature and grievous impact of hepatitis E infection on maternal and fetal life. In our study period, owing to an endemic, 32 patients delivered who were positive with a documented report of anti-HEV IgM positivity. Of these, the majority (78%) had not received adequate antenatal care hospital prior to registration at our hospital. The majority of women with jaundice, including those who were hepatitis E-positive, delivered vaginally. Amongst the pregnant women affected by hepatitis E, 12 (37.5%) presented with intrauterine demise in the antepartum period, and 2 (6.2%) had an intrapartum intrauterine demise. The rate of preterm delivery was 71.9%, and 28.1% delivered before 28 weeks. Seven (21.8%) women resulted in hepatic encephalopathy, and 31.2% had a deranged coagulogram. There were seven (21.8%) maternal deaths. All these numbers were significantly higher than in other types of viral hepatitis. Fetomaternal outcomes were significantly better with HBV, HCV, and HAV hepatitis compared with HEV, as shown in Table 3.

On applying the unpaired t-test, we found that adverse fetal outcomes were significantly associated with rising serum bilirubin levels of more than 11 mg/dL, serum glutamic oxaloacetic transaminase (SGOT) and serum glutamic pyruvic transaminase (SGPT) of more than 1000 IU/L, and a platelet count of less than 85.000 cells/mm^3^, and this difference was statistically significant.

Maternal outcomes were also significantly poorer in cases of serum bilirubin of more than 14 mg/dL, SGPT more than 1600, and SGOT more than 1200 and platelets less than 59.000 cells/mm^3^, as shown in Tables 5 and 6.

Looking at the fetomaternal outcomes at the time of delivery and applying the chi-square test, we found that preterm vaginal delivery had a statistically significant risk of poor fetal outcome, whereas it is was nonsignificant as far as poor maternal outcomes were concerned, as shown in Tables 7 and 8.

Similar poor outcomes have been reported by several authors. Sahai et al.^(11)^ reported that viral hepatitis in pregnancy led to high maternal mortality and also fetal wastage, especially if women presented with features of encephalopathy, fulminant hepatic failure, and coagulopathy.

Shinde et al.^(12)^ reported in their 2-year prospective study that 46.1% of pregnant patients developed encephalopathy compared with 34% in the non-pregnant group. Among the pregnant women, 67.3% survived and 32% died. In the non-pregnant group, most patients survived and only 9% died. This difference was statistically significant (p<0.01). Adverse fetal outcomes were seen in 71.1% of pregnant women with acute hepatitis E, including pre-term delivery in 23%, stillbirth in 23%, abortion in 3.8%, and intrauterine fetal death in 21.1% of patients.

Sultana and Humayun^(13)^ reported in their two years’ experience of 25 patients who had acute hepatitis E while one amongst them also had coexistent acute hepatitis A. Twenty-four hepatitis E positive (96%) patients presented in third trimester of pregnancy while one (4%) pregnancy ended in the second trimester as a missed miscarriage. Twenty-one (84%) babies were born alive, 18 (86%) of which were preterm. Perinatal mortality was 26%, which was contributed to by intrauterine deaths and early neonatal deaths in 3 (14%) cases each. In total there were 5 (20%) maternal deaths, 4 (80%) in postpartum period and 1 (20%) in the antepartum period due to fulminant hepatic failure.

A recent six-month study conducted by Singla et al.^(14)^ reported 27 HEV-positive (36%) women amongst a total of 82 women with jaundice. All five maternal deaths that they reported were not registered anywhere for antenatal care prior to presenting at their hospital and had raised bilirubin of more than 15 mg/dL with deranged coagulograms, encephalopathy, and intrauterine fetal deaths. On analyzing the morbidity data, it was also found that HEV-positive women had poorer outcomes as compared with their hepatitis B surface antigen-positive counterparts. In a 3-year prospective study, Prasad et al.^(15)^ reported 55 symptomatic women who were anti-HEV IgM-positive with maternal mortality of 5% and one antenatal death. Similar to our study, they also found prematurity and preterm rupture of membranes as the most common fetal complication.

In our experience, we found that 56% of women had normal outcomes, whereas the disease turned virulent in the rest with maternal death in 21.8% and fetal demise in 77.7%. What the factors are that govern the virulence of the disease and why virulence is typically increased in pregnant states remain unanswered questions. Bi et al.^(16)^ found that pregnancy serum accelerated HEV replication by suppressing oestrogen receptors and type I interferon in the early stage of infection. A comparison has also been reported between the clinical and subclinical presentation of the disease in pregnancy so as to understand why the disease can sometimes be self-limiting while other times it is life threatening. Several concepts have been studied to look for the cause of heightened disease severity in pregnancy. Ramdasi et al.^(17)^ showed antibody-dependent disease severity and impaired immune response in pregnant women with a differential elevation of cytokines in clinical and subclinical hepatitis E disease. The study by Pal et al.^(18)^ compared immune parameters among pregnant women with acute hepatitis E versus non-pregnant patients with hepatitis E and found reduced production of T-helper 1 (Th1) cytokines and an increase Th2 cytokines in the pregnant HEV group, which indicated a peculiar pathogenesis in pregnancy.

It is thought that a super infection of hepatitis E in patients with hepatitis B worsens liver disease. Huang et al.^(19)^ performed a two-year study to find the incidence of HEV infection among HBV-infected pregnant women and found that HEV infection was not a common occurrence with hepatitis B. None of their 391 HBV-infected patients were anti-HEV IgM-positive. We also had only one patient with both HBV and HEV positivity; we found the patient did not have a very different course than others.

Vertical transmission and intra-family transmission has also been studied and is variously reported in literature. It was reported as low by Gu et al.^(20)^, whereas Krain et al.^(21)^ have raised doubts about it due to inadequate research in the area of vertical transmission of HEV from mother to child.

The present study illuminates various points including the immense burden of disease in peak season, the extent of virulence in the form of fetomaternal life, and the uncertainty of disease course. The strength of the study is in the large number of test-positive HEV-positive pregnant women whose outcomes were evaluated.

### Study Limitations

The weaknesses of the study were the non-evaluation of virus positivity in neonates, plus the non-availability of immediate liver transplant for women with fulminant liver failure who might have been saved. Also, only symptomatic HEV-infected women were studied and asymptomatic women were missed because universal testing was not possible even in the endemic season due to resource constraints. The reasons of varied disease nature is a topic of future research, also it is important to time delivery after a test is determined positive because of the high incidence of intrauterine demise and prematurity.

## CONCLUSION

Hepatitis E is a deadly fetomaternal disease. The disease picture shows immense variation from patient to patient, which requires further research so as to better ascertain which factors play a role. It accounts for a significant number of deaths and increases the maternal mortality rate of the country.

The World Health Organization (WHO) also recognizes the burden of the disease. Due to the lack of sufficient information on safety, immunogenicity, and efficacy in pregnant women, the present position statement of the WHO does not recommend use of vaccines in pregnant women, or children aged <16 years^(22)^.

Unlike other diseases such as HIV, it also has a feco-oral route of transmission and hence the disease burden can be lessened by ensuring better sanitation and provision of clean drinking water for pregnant women.

## Figures and Tables

**Table 1:**
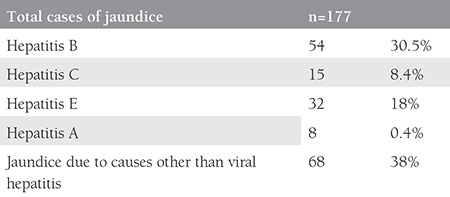
Different types of jaundice noticed in pregnant women

**Table 2:**
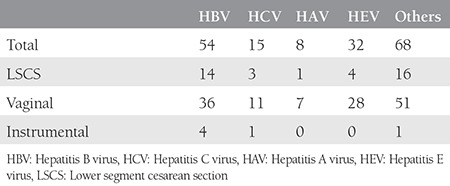
Mode of delivery in patients with jaundice in pregnancy

**Table 3:**
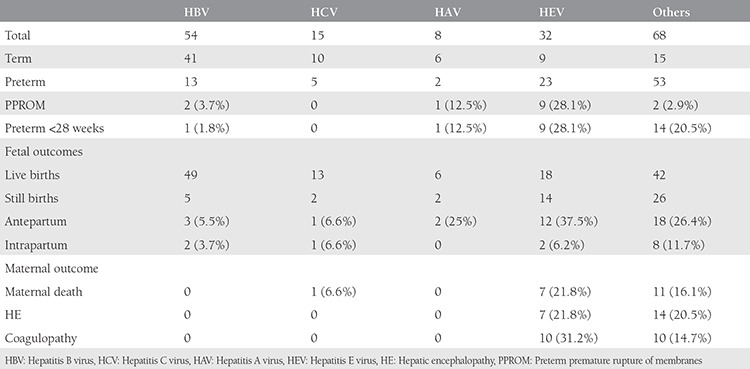
Distribution of different types of jaundice with fetomaternal outcomes

**Table 4:**
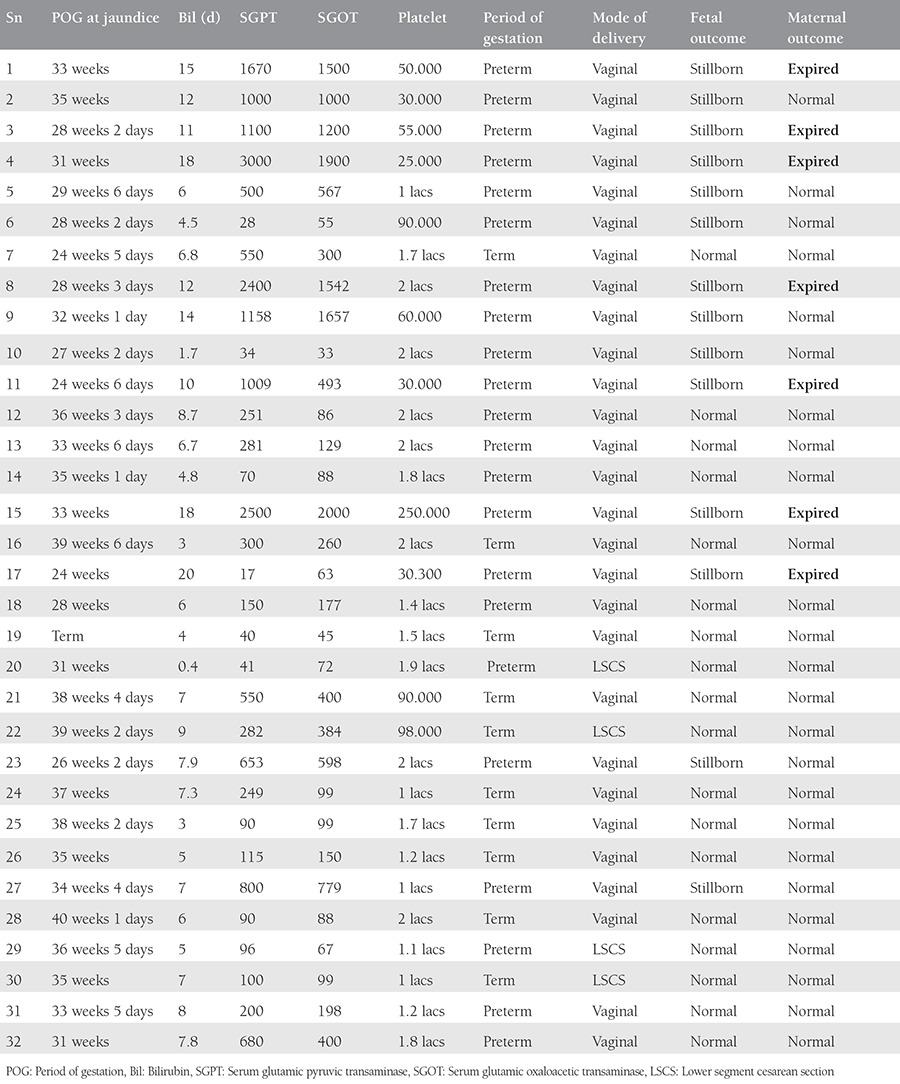
Details of hepatitis E infected cases

**Table 5:**
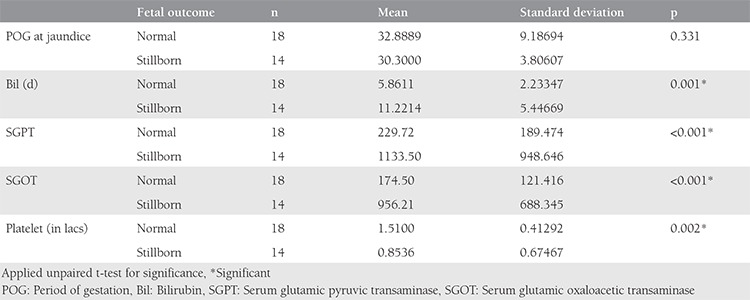
Comparison of various parameters with fetal outcome

**Table 6:**
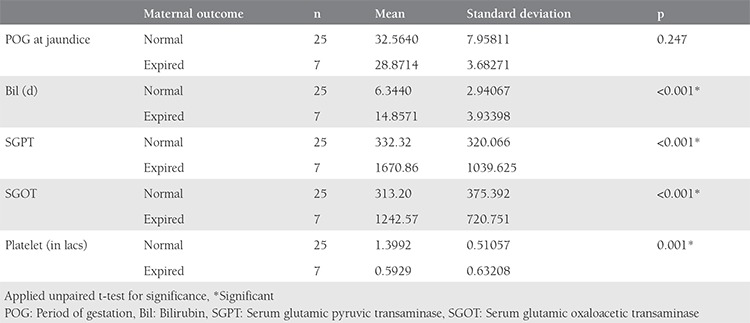
Comparison of various parameters with maternal outcome

**Table 7:**
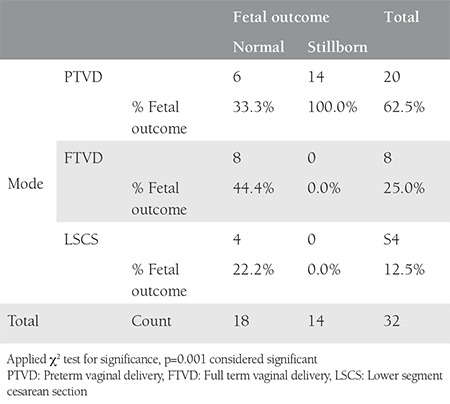
Relating mode and time of delivery with fetal outcome

**Table 8:**
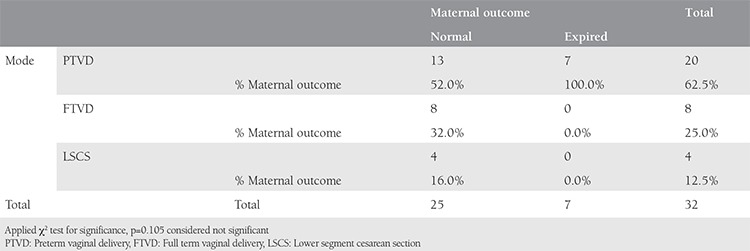
Relating mode and time of delivery with fetal outcome
